# Artificial intelligence in biology and medicine, and radioprotection research: perspectives from Jerusalem

**DOI:** 10.3389/frai.2023.1291136

**Published:** 2024-01-11

**Authors:** Yehoshua Socol, Ariella Richardson, Imene Garali-Zineddine, Stephane Grison, Guillaume Vares, Dmitry Klokov

**Affiliations:** ^1^Department of Electrical and Electronics Engineering, Jerusalem College of Technology, Jerusalem, Israel; ^2^Department of Data Mining, Jerusalem College of Technology, Jerusalem, Israel; ^3^Health and Environnent Division, Institut de Radioprotection et de Sûreté Nucléaire (IRSN), Fontenay-aux-Roses, France; ^4^Department of Biochemistry, Microbiology and Immunology, University of Ottawa, Ottawa, ON, Canada

**Keywords:** low doses, ionizing radiation, artificial intelligence, machine learning, radioprotection, public health

## Abstract

While AI is widely used in biomedical research and medical practice, its use is constrained to few specific practical areas, e.g., radiomics. Participants of the workshop on “Artificial Intelligence in Biology and Medicine” (Jerusalem, Feb 14–15, 2023), both researchers and practitioners, aimed to build a holistic picture by exploring AI advancements, challenges and perspectives, as well as to suggest new fields for AI applications. Presentations showcased the potential of large language models (LLMs) in generating molecular structures, predicting protein-ligand interactions, and promoting democratization of AI development. Ethical concerns in medical decision making were also addressed. In biological applications, AI integration of multi-omics and clinical data elucidated the health relevant effects of low doses of ionizing radiation. Bayesian latent modeling identified statistical associations between unobserved variables. Medical applications highlighted liquid biopsy methods for non-invasive diagnostics, routine laboratory tests to identify overlooked illnesses, and AI's role in oral and maxillofacial imaging. Explainable AI and diverse image processing tools improved diagnostics, while text classification detected anorexic behavior in blog posts. The workshop fostered knowledge sharing, discussions, and emphasized the need for further AI development in radioprotection research in support of emerging public health issues. The organizers plan to continue the initiative as an annual event, promoting collaboration and addressing issues and perspectives in AI applications with a focus on low-dose radioprotection research. Researchers involved in radioprotection research and experts in relevant public policy domains are invited to explore the utility of AI in low-dose radiation research at the next workshop.

## 1 Introduction

Biomedical research and medical practice are experiencing a revolutionary transformation driven by the integration of artificial intelligence (AI) into these fields. This is most evident for radiomics, a branch of medical imaging analysis, where the power of AI algorithms is harnessed to extract intricate information from medical images, such as MRI, CT scans, and X-rays to facilitate early disease diagnosis and personalized treatment planning (Bera et al., 2022). Beyond radiomics, AI plays an ever-increasing role in drug discovery, helping identify potential drug candidates and predict their efficacy (Jiménez-Luna et al., 2020). AI-driven genomics analysis aids in understanding complex genetic factors underlying diseases (Long et al., 2023). Moreover, AI assists in patient risk assessment, treatment optimization, and the development of precision medicine approaches, promising breakthroughs in healthcare (Xu et al., 2019). In public health, AI assisted approaches have been widely discussed in the last few years and their implementation is anticipated to drive significant progress (Thomasian et al., 2021). Radioprotection of the public aims to manage and control the health risks associated with the exposure to low doses of ionizing radiation, e.g., due to natural sources (radon, cosmic rays, terrestrial background radiation) and anthropogenic sources (diagnostic imaging, occupational exposure in nuclear industry). Progress in understanding biological effects and health consequences of exposure to low-dose ionizing radiation has been flawed with substantial inconsistencies and controversies (Feinendegen, 2020), underlying the importance of robust analyses of complex multi-modal and multi-omics datasets generated by animal studies. The use of AI in both radiation risk estimation and data analyses to unveil complex biology of low-dose radiation effects has been proposed but progress has been slow.

The International workshop “Artificial Intelligence (AI) in Biology and Medicine” was held at the Jerusalem College of Technology (JCT), Jerusalem, Israel on 14–15 Feb 2023. This “boutique” workshop was a two-day event precipitated as the outcome from a collaborative project between JCT and IRSN (Institute of Radioprotection and Radiation Safety, Fontenay-aux-Roses, France) focused on the use of AI for analyzing low-dose ionizing radiation effects on intestinal tumorigenesis in mice. We decided however to open this event to a broader research community since AI has been steadily finding its use in various studies in radiation biology (Wahid et al., 2022; Wilson et al., 2023) and medicine (Aceves-Fernandez, 2019). Twenty researchers and R&D experts from Israel, France and Germany presented their results (Table 1) and discussed with colleagues issues and perspectives. Fifteen JCT students also presented their results in nine posters.

**Table 1:** The workshop speakers and their areas of expertise.

**Speaker name**	**Affiliation^a^**	**Area of expertise**
Olivier ARMANT	IRSN	Computational biology
Ori ASHUSH	JCT	Computational pathology
Mohamed Amine BENADJAOUD	IRSN	Bioinformatics, biostatistics
Gavriel FIALKOFF	Hebrew University of Jerusalem	Bioinformatics
Imene GARALI ZINEDDINE	IRSN	Systems biology
Stephane GRISON	IRSN	Radiation biology
Yaakov HACOHEN-KERNER	JCT	Natural language processing
Dmitry KLOKOV	IRSN	Radiation biology
Benjamin MILGROM	JCT	Computer vision
Chen NADLER	Hadassah Medical Center	Computational pathology
Igor PECHERSKY	ANAGOG Ltd.	Big Data processing
Nadav RAPPOPORT	Ben-Gurion University	Big Data processing
Judith REINDL	Universität der Bundeswehr München	Radiation biology
Ariella RICHARDSON	JCT	Machine learning
Avi ROSENFELD	JCT	Explainable AI
Chen SAGIV	DeePathology Ltd.	Computational pathology
Yehoshua SOCOL	JCT	Statistics, modeling
Guillaume VARES	IRSN	Molecular biology
Moshe YANOVSKIY	JCT	Ethics
Talia YESHUA	JCT	Computational pathology

^a^IRSN, Institut de Radioprotection et de Sûreté Nucléaire; JCT, Jerusalem College of Technology.

The workshop commenced with an opening address from Prof. Chaim Sukenik, the JCT President followed by Prof. Avi Domb, the Chief Scientist of the Israeli Ministry of Innovation, Science and Technology, and Mrs. Camille Padet, the Scientific Officer of the French Embassy in Israel. In this article, we provide a structured summary of the presentations, not in any particular order of importance or chronology [more information at (AIBM, 2023)]. In concluding remarks, we provide opinion on the perspectives of the development of the AI algorithms in the domain of low-dose radiation effects research and how it may contribute to the radioprotection of the public within a general area of public health.

## 2 Presentations

### 2.1 General

Igor Pechersky presented foundation models, also known as large language models (LLM). These are large-scale models—some with over 500 billion parameters—trained on vast amounts of unlabeled data using self-supervision. LLMs have demonstrated remarkable capabilities that rival human performance on complex tasks, such as answering difficult questions, generating coherent contexts, summarizing ideas, reasoning, and planning. More recently, studies have shown that these systems can successfully generate new molecular structures with desired properties, predict protein-ligand interactions, and identify potential drug targets (Guo et al., 2023). LLMs also adapt to the users' prompt, a feature that has led to the emergence of a new demanded job: the AI prompter or “LLM psychologist.” Pechersky highlighted an important feature of the current trend in the AI field: “democratization” (Cohen, 2023). This means that libraries, models, data, and computation resources are becoming increasingly available, fostering thriving communities and collaboration.

Chen Sagiv approached the concept of “democratization” from a different perspective and discussed the shift in the paradigm in AI development: from AI being simply a tool to becoming a playground for practitioners. Traditionally, practitioners (such as medical doctors) present problems that AI experts attempt to solve. However, a recent trend in AI empowers practitioners to develop AI solutions for their specific domains through accessible platforms and tools. This new paradigm allows practitioners to explore AI solutions for daily tasks and develop these solutions independently.

Moshe Yanovskiy highlighted ethical challenges of using AI in medical decision making. While some of these issues, such as the “moral hazard” of asymmetric information (AI developer—AI developer's expert—medical practitioner—patient), have been discussed for decades, others are more specific to AI (Zheng et al., 2019; Cohen, 2023). Practitioners' fear of competition or unemployment (a modern form of Luddism), can jeopardize proper deployment of AI systems. However, a more pressing and specific ethical concern is the potential for developers and regulators to promote their subjective choices under the veil of anonymity, thereby imposing them on practitioners and ultimately on patients. This can result in unforeseen and grave consequences (Yanovskiy and Socol, 2023a). For example, including equity as a component of an AI utility function may force AI decision that might decrease the survival rate of some human cohorts (being unable to increase survival rate of other cohorts). These and other challenges have led to calls for strict government regulation of AI, but since the government is neither an error-free machine nor a heavenly host (Yanovskiy and Socol, 2023b), caution must be exercised in implementing such standards.

### 2.2 Biological applications of AI

Dmitry Klokov summarized the results of the IRSN–JCT collaborative project which investigated various AI approaches to integrating multi-omics and clinical data to understand the effects of low doses of ionizing radiation on intestinal tumorigenesis in the APC^Min/+^ mouse model. It was highlighted that the study encountered several challenges that are common in preclinical biomedical research, such as data heterogeneity, missing values, confounding, and small sample size. The Similarity Network Fusion method (SNF) that was shown to be efficient for the integration of different data modalities (Wang et al., 2014) revealed that the effects of low doses were overshadowed by the effect of age, underscoring the need for innovative data integration approaches to improve sensitivity. To this end, several supervised and unsupervised multivariate approaches were tested, with CCA-MaxVAR (Vía et al., 2007) demonstrating the best performance. The author emphasized the biological relevance of the results of multi-omics analyses, revealing the role of immunological and inflammatory pathways consistent with clinical observations. The presenter also highlighted the potential applicability of this approach to other low-dose radiation effects studies and the importance of forward-thinking in planning such studies.

Olivier Armant presented on the long-term evolutionary impact of the Chernobyl nuclear accident on tree frog (*Hyla Orientalis*) populations and on the use of multi-omics data analyses using AI. Although the wildlife in the Chernobyl exclusion zone is commonly believed to be thriving, there are also concerning indicators (Car et al., 2022). In the case of *Hyla Orientalis*, lower effective population size and deteriorated body condition were observed in populations that were most exposed to ionizing radiation. Furthermore, through the use of both supervised and unsupervised AI methods, the authors detected a distinct transcriptomics signature and stop-gained mutations in genes involved in energy metabolism (Car et al., 2023).

Mohamed Amine Benadjaoud presented a Bayesian latent modeling approach for assessing statistical association between variables that are not jointly observed. In biology, obtaining a “single-source dataset” where all variables are measured from the same animal (e.g., gene expression + inflammatory markers + organ pathology) is often difficult or impossible, as many observations require animal sacrifice or mutually exclusive. The lecturer presented an overview of several approaches based on Bayesian statistics and latent variable modeling which constrain the causal mechanism underlying the observations collected in multiple datasets. The proposed approach was illustrated in two studies: a study demonstrating the beneficial effect of exosomes derived from human Mesenchymal Stem Cells on the survival of mice with radiation-induced gastrointestinal syndrome and a second study investigating biomarkers of subclinical cardiac dysfunction in a cohort of rats irradiated with several doses to the heart (Ribeiro et al., 2022).

Judith Reindl presented a system for intelligent detection, tracking and cell cycle evaluation of eukaryotic cells on videos generated using phase-contrast live-cell microscopy. The system harnesses the power of CeCILE (Cell Classification and *In-vitro* Lifecycle Evaluation), a deep-learning based algorithm that uses a faster RCNN (Region-based Convolutional Neural Network) (Rudigkeit et al., 2021). RCNN algorithm identifies potential object regions in an image using selective search and then applies a convolutional neural network to classify each region. This two-step process allows it to accurately detect and localize multiple objects in an image, combining region proposals with deep learning. The model was trained on a hand-labeled dataset of microscopic videos, allowing the authors to investigate the behavior of irradiated cells in a simple experiment and to obtain results that were comparable to state-of-the-art assays. By including a tracking method, they were able to go even further, examining the behavior of every single cell and evaluating the cell-cycle, lineages and circumstances of cell death.

A dedicated session was held for the IRSN–JCT “Maïmonide-Israel” project, during which the authors of this paper presented their findings. While the project was briefly discussed earlier, it is worth noting that subsequent stimulating discussions, involving participants outside the project, highlighted new, promising approaches. For example, utilizing the CCA-MaxVAR identified genes as an input layer in a neural network may make deep learning algorithms more suitable for small sample size datasets. Additionally, integrating datasets acquired from different sources, such as tumor scores and RNAseq data from separate subjects/animals, can be addressed through Bayesian statistics and latent variable modeling when a third common dataset is available. The study's results will be soon reported elsewhere.

### 2.3 Medical applications of AI

Gavriel Fialkoff's and Nadav Rappoport's presentations successfully bridged the fields of biology and medicine, while other presentations focused on AI applications in medical practice. Gavriel Fialkoff's topic “Deconvolution of cell-free nucleosomes and inferring tissue-of-origin and transcription programs in autoimmune hepatitis plasma” explored liquid biopsy methods as a powerful non-invasive diagnostic and disease monitoring tool applicable in several pathological contexts. While most bioinformatics diagnostic methods that use cell-free nucleosomes rely on identifying nucleotide sequence changes, recent approaches aim to identify the tissue-of-origin without such changes by analyzing DNA methylation, fragmentation patterns (Oberhofer et al., 2022). A recently published method cfChIP-seq for chromatin immuno-precipitation and sequencing of cell-free nucleosomes holds promise in inferring transcriptional programs taking place in dying cells (Sadeh et al., 2021). The author discussed the considerations involved in deconvoluting cell free nucleosomes, proposed a Poisson regression model for elicidating cell-type composition and transcription programs, and applyied this model to cfChIP data from autoimmune hepatitis patients.

Nadav Rappoport demonstrated that routine laboratory tests can be a valuable tool for identifying patients at risk for overlooked illnesses, provided that multidimensional data analysis is performed. The authors used data from the UK Biobank (~500,000 subjects) and the Mount Sinai Data Warehouse (~100,000 subjects), and analyzed about 30 parameters obtained from routine laboratory tests, such as complete blood counts, chemistry and urinalysis. They achieved an accuracy rate of ~75%. Moreover, the authors estimated the biological age of patients using an XGBoost model—an efficient and scalable machine learning algorithm for gradient boosting, known for its performance and speed in classification and regression tasks—based on data from about 472,000 individuals aged between 37 and 82. The model was able to accurately predict the biological age of subjects, with those predicted to be younger than their physical age being found to be healthier. On the other hand, subjects predicted to be biologically older did not exhibit significant differences when compared to age-matched controls.

Avi Rosenfeld discussed how explainable artificial intelligence (XAI)—a set of processes and methods that allows human users to comprehend and trust the results and outputs generated by machine learning algorithms—can be quantified and applied for improved AI safety in medicine. The authors developed four metrics for quantifying XAI: (1) the D metric which is based on performance differences between the explanation's logic and the system's actual performance, (2) the R metric which quantifies the number of rules outputted by the explanation, (3) the F metric, which quantifies the number of features used to generate that explanation, and (4) the S metric, which quantifies the stability of the explanation. The presentation included a specific example of how all four metrics can be applied to better quantify AI safety to improve detection of several types of cancer.

Chen Nadler presented on the use of AI in oral and maxillofacial imaging at Hadassah Medical Center, Israel. The study included 41 dental cone beam CT (CBCT) scans with, and 41 without histologically confirmed benign jaw lesions from 3 different CBCT machines (Yeshua et al., 2023). The team utilized MASK-RCNN to detect the bone lesion in each axial section. The developed algorithm was able to detect all 11 scans with bone lesions, with 100% accuracy. The sensitivity ranged from 82.0% to 100%, with a total sensitivity of 95.9%, and the final precision ranged from 92.4% to 100%, with a total precision of 98.8%.

Talia Yeshua presented on the use of diverse image processing and machine learning tools for automatic diagnosis of medical and biological images, in collaboration with researchers and physicians from various departments including radiology, orthopedic surgery, urology, otorhinolaryngology, microbiology, and oral medicine. The role of the presenter's laboratory is to determine the most appropriate AI tools to achieve optimal automatic diagnosis. During the lecture, some of the tools used for automatic diagnosis were briefly reviewed and the advantages of combining several AI tools for the best diagnostics were demonstrated.

Yaakov HaCohen-Kerner's presented on the “Detection of anorexic girls in blog posts written in Hebrew using a combined heuristic AI and NLP methods”. Natural Language Processing (NLP) is a field of artificial intelligence that focuses on enabling computers to understand, interpret, and respond to human language in a way that is both meaningful and useful. The authors constructed a dataset of blog posts written by females who either exhibited (100 posts) or not (100 posts) likely anorexic behavior. Then, they tested several text classification (TC) methods, using various feature sets (content-based and style-based), five classical machine learning (ML) methods, three RNN models (Recurrent Neural Network (RNN) is a type of artificial neural network designed to recognize patterns in sequences of data, such as text, genomes, handwriting, or spoken words, by processing inputs with its internal memory), four BERT models [(Bidirectional Encoder Representations from Transformers) is a groundbreaking method in natural language processing pre-training that learns to understand the context of a word in a sentence by looking at the words that come before and after it], three basic pre-processing methods, three feature filtering methods, and parameter tuning. A heuristic process based on the random forest ML method has overcome a combinatorial explosion and led to significant improvement over a baseline result at a significance level of P = 0.01. Additionally, an iterative process was applied, testing various combinations of features, boosting performance to an accuracy score of 91%, using a combination of 300 features from 10 feature sets.

Ori Ashush's presentation focused on feature harmony classification for the detection of handwriting disorders. Instead of using a subset of features to characterize specific problems, the authors explored the entire set of handwriting parameters to determine its overall harmony and smoothness in multiple parameters. The team utilized random forests and neural nets on various datasets of handwriting captured on a Wacom tablet, including those from children and adults with DCD, dysgraphia, Parkinson's, and Alzheimer's.

Benjamin Milgrom discussed the use of autoencoders in depth sensing for vision enhancement by increasing the depth of field of the imaging system through the use of a coded phase mask (Milgrom et al., 2020). The improvement was achieved by combining image processing algorithms, based on autoencoders. An added value of the improvement is passive range estimation, which defines areas of interest in the image according to the distance from the lens. The presented algorithm is tailored for machine vision applications and can improve the orientation of the visually impaired by means of an affordable and simple-to-operate component.

## 3 Summary and perspectives

The workshop provided an excellent opportunity to meet, share results and discuss pressing issues and emerging ideas related to the use of AI in biology and medicine. It is an opinion shared by all workshop participants that the objectives of the workshop were successfully achieved. While the topics presented and discussed during the workshop were diverse, as well as expertise profiles of the participants, ensuing discussions proved the importance of such inter-disciplinary scientific forums to stimulate “outside-the-box” thinking and identify new promising avenues and perspectives. The poster session enabled 15 young investigators from JCT to present their work and engage in discussions with established experts, an important contribution to generating new young researchers in the field of AI in biology and medicine.

The workshop highlighted that the integration of AI in medical applications has been substantially stronger compared to biological and biomedical research. This gap is particularly large for the domain of radiological protection research, a domain that encompasses two major disciplines: epidemiological studies of radiation health risks on radiation-exposed human cohorts and laboratory studies of low-dose radiation induced changes in cells, tissues and organisms *in vivo* that are relevant to adverse health outcomes. These studies are essential for generating scientific support for the assessment of radiation health risks of the public, patients and radiation exposed professionals, such as medical personnel and nuclear energy workers. However, both radiobiology and radioepidemiology have their own specific limitations. Epidemiological studies suggest an association concept rather than a causation approach and mechanistic insight, while *in vivo* animal studies are challenged with difficulties in interpreting the complex data in the context of such multifactorial complex diseases as cancer and cardiovascular disease. The use of AI can then facilitate the integration of findings from both disciplines.

The work of a joint IRSN-JCT team presented at the workshop explored promising approaches for such integration. Other issues in low- and high-dose experimental radiobiology and radiotoxicology research include small group size and data heterogeneity that affect the ability to extract information on biological mechanisms influenced by low doses relevant to health outcomes. Such use of AI not only enables the integration of such complex datasets, but can also facilitate the development of Adverse Outcome Pathways (AOPs) for the estimation of radiation public health risks and knowledge gap identification (Chauhan et al., 2021). Another promising use of AI for the development of radiation AOPs is the use of text mining algorithms to collect and consolidate supporting experimental evidence in literature (Jaylet et al., 2022). These examples of AI applications in advancing the public radiation risk assessment hold promise but require further investigation and development.

Optimal AI application in radioprotection research necessitates the management, availability, sustainability and sharing of comprehensive data, underscored by repositories like the OECD NEA's Global Register of Low-Dose Radiation Projects (GR-LDR, 2023) and databases detailed by Zander et al. (2019). These platforms provide AI developers with diverse data essential for effective training and research. Accessible data from such repositories can be a starting point for building collaborative data ecosystem and for integrating mechanistic laboratory findings with radiation-exposed human cohort studies to empower AI to improve radiation health risk assessment.

To continue the momentum generated in Jerusalem, the organizers plan to develop this workshop into an annual event, where pressing and emerging issues related to the use of AI in biomedical research can be discussed in a welcoming and exchange-facilitation atmosphere. The identified gap in the readiness of AI approaches in radioprotection research (Laurier et al., 2021; Burtt et al., 2022), presents an opportunity for the next workshop to focus on the utility of AI in low-dose radioprotection research and development. The organizers believe and hope that this focus can generate sufficient interest among the research community, particularly given the expertise of the core group of organizers in radiation protection research. To this end, they invite readers involved in radioprotection research, including radiobiology, epidemiology, environmental ecotoxicology, dosimetry, social studies, and other related disciplines, who have developed an interest in using AI in their research, to contact the authors with respect to participation in the next workshop.

## Data availability statement

The original contributions presented in the study are included in the article/supplementary material, further inquiries can be directed to the corresponding authors.

## Author contributions

YS: Conceptualization, Project administration, Writing–review & editing, Writing–original draft. AR: Writing–review & editing. IG-Z: Writing–review & editing. SG: Writing–review & editing. GV: Writing–review & editing. DK: Conceptualization, Project administration, Writing–review & editing.
